# Logical Resolving-Based Methodology for Efficient Reliability Analysis

**DOI:** 10.3390/mi15010085

**Published:** 2023-12-30

**Authors:** Zhengguang Tang, Cong Li, Hailong You, Xingming Liu, Yu Wang, Yong Dai, Geng Bai, Xiaoling Lin

**Affiliations:** 1 School of Microelectronics, Xidian University, Xi’an 710071, China; zgtang@stu.xidian.edu.cn; 2 SMiT Group Fuxin Technology Limited, Shenzhen 518000, China; liuxm@gwxeda.com (X.L.); wangyu@gwxeda.com (Y.W.); ydai@smit.com.cn (Y.D.); gbai@smit.com.cn (G.B.); 3 The Science and Technology on Reliability Physics and Application of Electronic Component Laboratory, China Electronic, Product Reliability and Environmental Testing Research Institute, Guangzhou 510610, China; lin_x_l@163.com

**Keywords:** reliability simulation acceleration, bias temperature instability (BTI), stress probability evaluation, static timing analysis

## Abstract

With the CMOS technology downscaling to the deep nanoscale, the aging effects of devices degrade circuit performance and even lead to functional failure. The stress analysis is critical to evaluate the influence of aging effects on digital circuits. Some related analytical work has recently focused on reliability-aware circuit analysis. Nevertheless, the aging dependence among different devices is not considered, which will induce errors of degradation evaluation in the digital circuit. In order to improve the accuracy of reliability-aware static timing analysis, an improved analytical method is proposed by employing logical resolving. Experimental results show that the proposed method has a better evaluation accuracy of aging path delay than traditional strategies. For aging timing evaluation on aging paths, excessive pessimism can be reduced by employing the proposed method. And, a 378× speedup is achieved while having a 0.56% relative error compared with precise SPICE simulation. Moreover, the circuit performance sacrifice of an aging-aware synthesis flow with the proposed method can be decreased. Due to the high efficiency and high accuracy, the proposed method can meet the speed demands of large-scale digital circuit reliability analysis while achieving transistor simulation accuracy.

## 1. Introduction

With the rapid technology scaling of CMOS circuits, the performance degradation induced by the transistor aging is more evident, which leads to a more serious negative influence on the circuit [1]. Aging effects like positive bias temperature instability (PBTI) and negative bias temperature instability (NBTI) degradation [2] cause the threshold voltage of transistor to shift ( Δ *V*_th_) and worsen the performance of circuit [3]. Especially, the BTI effect will degrade the digital circuit delay by up to 20% over ten years [4]. In order to mitigate the negative influence of BTI aging on digital circuits, it is critical to accurately evaluate the aging results of all devices in the circuit.

**Figure 1:**
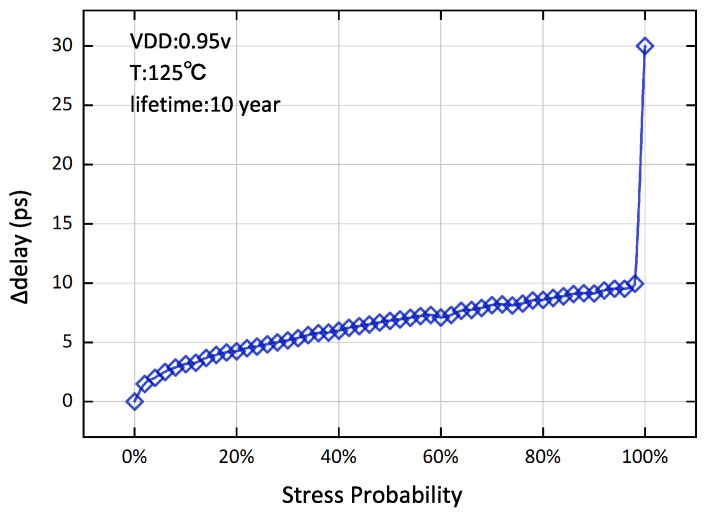
The delay difference (aged delay—fresh delay) of FO4 under both NBTI and PBTI aging. The PTM model [7] is used and the aging model will be given in Section 4.1.

BTI-induced degradation in transistors, involving trap generation and accumulation in the oxide layer due to BTI stress, can experience partial recovery during stress-free periods. This recovery is associated with trap redistribution and electron release processes in the oxide layer, allowing for the release of previously trapped electrons and mitigating the increase in trap density. The degradation caused by BTI aging is mainly determined by aging stress conditions like temperature, voltage, and stress probability (SP) which represents the ratio of stress time during the lifetime [5]. Among these conditions, SP dramatically varies the degradation of device parameters such as Δ *V*_th_ at the same lifetime [6]. Therefore, accurately evaluating SP is essential to predict aging delay for digital circuits. Take fan-out-of-four (FO4) (fan-out of 4 is generally used as a delay metric, where one FO4 is the delay of an inverter which simultaneously drives four other inverters) in Figure 1 as an example. An optimal case is that SP equals “0”, meaning that the device will have no degeneration of threshold voltage, so the difference between the aging and fresh delay will be 0.

When SP is equal to “1”, the device will be frequently stressed throughout its lifetime and the timing degradation will significantly increase, as shown in the trend at the end of the curve in Figure 1. The reason is that the aging defects are partially healed during stress-free phases, but the recovery will be just enough to ignore as the SP approaches “1” [8]. Therefore, accurately evaluating SP is essential for aging analysis on the digital circuit [9,10].

To quantify the SP of aging devices, the most precise approach is directly employing a SPICE simulation for the aging circuit [1]. In detail, the reliability simulation tool performs transient simulation at the transistor level. It is achieved by accumulating the degradation value for every transient simulation step and extrapolating to the lifetime [11]. Despite the fact that the transistor-level simulation can achieve precise SP evaluation, the transistor-level simulation process will take much computing power. On the one hand, restoring all signal waveforms in the very large-scale integration (VLSI) circuit design will bring a memory explosion [12]. On the other hand, it will take much time to apply SPICE in an aging simulation. Although simulation acceleration can be achieved by relying on the parallel acceleration method [13], it still requires massive computing resources. For the above two reasons, the analytical strategy is developed to evaluate SP results with less time and memory consumption [9]. Among these analytical methods, the state-of-the-art work [9,14,15,16] calculates the SP values according to logical function or internal structure of std-cells. The published analytical work can achieve a certain degree of accuracy while significantly reducing the computation consumption, but the aging dependence among different devices was neglected. Through further research, we found that neglecting the aging dependence will lead to an evident loss of accuracy for SP analysis. Furthermore, as mentioned previously, inaccurate SP evaluation will mislead the aging analysis for digital circuits. In order to show the influence of aging dependence among different aging devices and improve the accuracy of SP results, we propose an improved analytical method based on logical resolving to evaluate SP results.

**Figure 2:**
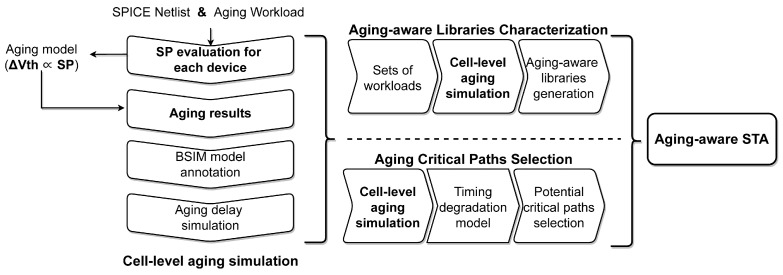
The aging STA flow is based on analytical SP calculation. Note that SP is one of the parameters in the long-term aging model (see Section 4.1), and the aging result Δ *V*_th_ is obtained based on SP.

Additionally, the proposed method is combined to verify the benefit for subsequent aging-aware static timing analysis (STA) flows. As shown in Figure 2, the flow of SP evaluation is the basis of aging-aware STA [17]. At first, the threshold voltage degradations of MOSFETs inside the std-cell are calculated based on SP results and the aging model. And then, the aging delay of a single std-cell is obtained by annotating the BSIM parameter and employing the SPICE simulation. The above two steps constitute the cell-level aging simulation, which is indispensable for aging timing analysis [1]. Based on the cell-level aging simulation, it can be extended to the aging-aware STA for digital circuits, whose typical practices include aging-aware std-cell libraries characterization [18] and distinguish the potential critical paths [19]. So we will verify both two practices combined with the proposed methods. Through the effective verification process for the aging-aware STA flows by incorporating the proposed method, the refined flows can provide a better reference for aging-aware digital circuit design.

The main contributions of this paper are as follows:

1. We raise and discuss the effect of aging dependence on SP evaluation. A logical resolving-based approach is proposed to consider the aging dependence. Moreover, the accuracy of the aging path analysis is verified by experimenting on ISCAS’85 benchmarks.
2. Compared with previous work, the proposed method combines aging-aware library characterization at the cell level to mitigate pessimistic predictions and better capture the correlation between aging and stress conditions.
3. Because our results are closer to simulation and not overly pessimistic, the aging-aware synthesis flow with the proposed method will avoid extra design margin loss. This improvement benefits the circuit design flow that considers aging margins early in the design process.

The remaining part of this paper is organized as follows. Section 2 presents the background and motivation for our work. Section 3 describes the proposed method for extracting aging dependence and solving the aging function of individual devices. Our experimental results and related analysis are shown in Section 4. Finally, the conclusion is given in Section 5.

## 2. Background

### 2.1. Preliminaries

To transfer the aging condition of the BTI effect to signal logical relationships on digital circuits, we will first introduce the BTI physical mechanism and give the terminal logic combination for stressed MOSFET. Second, some assumptions and simplifications adopted in this paper will be given.

BTI aging in digital circuits. BTI is a common reliability issue in modern semiconductor devices. It refers to the gradual degradation of device performance over time, caused by the trapping and release of charge carriers at the interface between the semiconductor and the insulating layer. There are two main types of BTI: NBTI (negative BTI) and PBTI (positive BTI). NBTI occurs when the negative voltage stress is applied to a PMOS, causing electron trapping in the gate dielectric layer. PBTI occurs when positive voltage stress is applied to a NMOS, causing hole trapping in the gate dielectric layer. Both NBTI and PBTI can significantly impact the reliability and performance of semiconductor devices, particularly as CMOS technology continues to downscale and operating voltages increase.

When the gate terminal is biased to enable device conduction, and the source (drain) terminal is charged to a logical “1” or discharged to a logical “0”, the drain (source) terminal will transition to the same logical value. This process can cause the BTI aging effects [20]. In summary, the aging conditions of NBTI and PBTI are summarized as Equation (1):

(1) $$\begin{array}{c} \left\{\begin{array}{c} \begin{array}{c} NBTI\text{\_}contidion:\;(gate=“0”)\;\&\&\;(source=“1”\;∥\;drain=“1”)\\ PBTI\text{\_}contidion:\;(gate=“1”)\;\&\&\;(source=“0”\;∥\;drain=“0”) \end{array} \end{array}\right. \end{array}$$

where “0” presents logical “0” and “1” presents logical “1”. And in this paper, we use the PL symbol for the duty cycle of the logical “0” and the PH symbol for logical “1”.

Assumptions and simplifications. Considering the conclusions from previous works [9,15,20,21], we make the following assumptions and simplifications for BTI aging analysis:

1. Although BTI aging is present in combinational and sequential cells in the std-cell library, sequential cell degradation has little impact on the total path aging delay. In contrast, combinational cells dramatically vary in their critical path delay [15,21]. So, in this paper, only combinational cells are considered. And, all sequential cells are set into the worst-case aging scenario [21].
2. As shown in Equation (1), the aging condition is related to the switching state and duty cycle between the signals. A previous work [20] also illustrates that there is spatial and temporal relevance for the signals inside the circuit. However, recent work [9] has demonstrated that the relevance within the inputs of the std-cell has little impact on aging analysis. Therefore, all input signals of each cell will be treated as independent (i.e., switching one signal will not affect the state of another).

Additionally, the SPICE aging simulation is evaluated based on real stress without simplifying and assuming, so the SPICE aging simulation is treated as golden.

### 2.2. State-of-the-Art and Their Lack

Based on an example, this subsection will introduce the implementation detail of state-of-the-art analytical work [9,14,15,16] and their lack of considering the aging dependence among different devices. The related work [9,14,15,16] on SP characterization is based on the assumptions and simplifications provided in Section 2.1. They involve solving the degradation probabilities for each individual device according to the circuit structural information. Nevertheless, all the above characterization methods ignored the dependent aging relationship among the different devices. This will influence the evaluation accuracy to a considerable degree. Moreover, these aging result errors of devices will propagate and enlarge in the cell-level and circuit-level aging evaluation flows.

**Figure 3:**
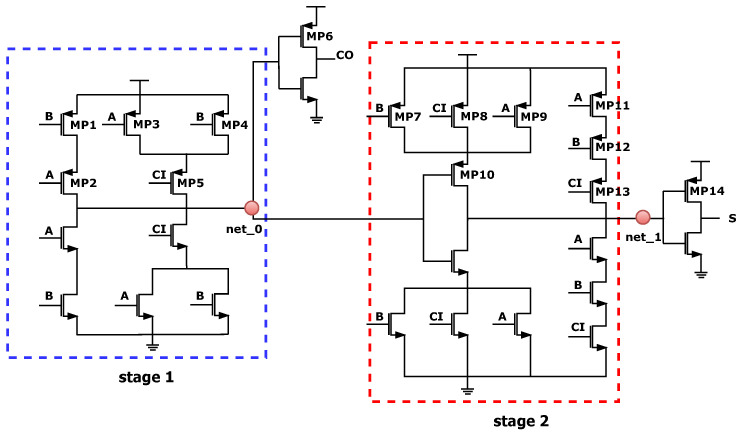
Circuit-level schematic of a full adder. Where *A*, *B*, and CI are input signals and C0 and *S* are output signals. The stages are split based on the standard of intermediate signal serving as the output or driving signal.

To prove that aging dependence needs to be considered, the derivation process of conventional methods [9,14,15,16] mentioned above and the analysis considering aging dependence will be given. Take NBTI as an example, the implementation steps of a conventional method are summarized as follows. First, multiply the cut-off probability of every stress path, where the path is defined as the conducting path charging the source or drain terminal to logical “1”. Second, subtract by a constant of one, which means that the probability of at least one stress path conducting. And then, multiply the probability of the gate signal in logic “0”. For a more detailed explanation, as shown in Figure 3, for *MP*5, three paths may hold MP5 under stress: MP1(on) & MP2(on) , MP3(on) , MP4(on) . The aging relationship of MP5 is symbolized as

(2) $$\begin{array}{cc} SP(MP5)=\; & PL(CI)\;\&\;(\;PL(A)\;|\;PL(B)\;|\;(PL(A)\;\&\;PL(B))\;) \end{array}$$

According to conventional methods and the assumption that all cell input signals are independent, the result would be

(3) $$\begin{array}{c} SP(MP5)=PL(CI)*(\;1-PH(A)\;*\;PH(B)*(1-PL(A)*\;PL(B))\;) \end{array}$$

While the result considering the dependent relationship among devices should be

(4) $$\begin{array}{cc} SP(MP5) & =\;PL(CI)*(\;1-PH(A)*PH(B)\;)\\ \; & =PL(CI)*PL(A)+PL(CI)*PH(A)*PL(B) \end{array}$$

Compared with Equation (3), the additional multiplex item in Equation (3) will induce a subtracted item containing the duplicating event for (1 − PL(A)&PL(B) ). The duplicating event would make the SP result of MP5 more pessimistic.

And, for MP10 in Figure 3, it is more complicated to be considered the intermediate signal net_0 between adjacent stages. First, the PL of signal net_0 needs to be obtained from the pull-up and pull-down network. Similarly, if all paths and signals are assumed to be independent of each other, the related probability is expressed as

(5) $$\begin{array}{cc} \; & \mathrm{anyone}\;\mathrm{pull}\text{-}\mathrm{down}\;\mathrm{path}\;\mathrm{conducts}\text{:}\\ \; & PL(net\text{\_}0)=1-(1-PH(A)\cdot PH(B)\cdot (1-PH(CI)\cdot PH(A))\cdot (1-PH(CI)\cdot PH(B)))\\ \; & \mathrm{all}\;\mathrm{pull}\text{-}\mathrm{up}\;\mathrm{paths}\;\mathrm{cut}\text{-}\mathrm{off}\text{:}\\ \; & PL(net\text{\_}0)=(1-PL(A)\cdot PL(B))\cdot (1-PL(A)\cdot PL(CI)\cdot (1-PL(B)\cdot PL(CI))) \end{array}$$

In fact, the two above equations also contain repeat probabilistic events, and the related outcomes vary. Consequently, for MP10 , the conventional approach will obtain more errors without considering the driving gate signal dependence. We choose the condition of anyone pull-down path conducting as the comparison object in the following discussion. Similarly to the result of MP5 , any one stress path conducting the probability of conventional methods would be

(6) $$\begin{array}{cc} \; & 1-PH(A)*PH(B)*PH(CI)*(1-PL(A)*PL(B)*\;PL(CI)) \end{array}$$

Similarly to Equation (4), Equation (6) neglects independent relationships among different stress paths, which include the duplicating event for PH(A)&PH(B)&PH(CI) . Then, multiplying Equations (5) and (6) will cause more errors in SP(MP10) . While the precise equation with the consideration of aging dependence should be expressed as

(7) $$\begin{array}{c} SP(MP10)=PL(A)*PH(B)*PH(CI)+PH(A)*\\ PL(B)*PH(CI)+PH(A)*PH(B)*PL(CI) \end{array}$$

The above investigation indicates that conventional methods would introduce extra errors from two aspects: the dependence among aging paths; the correlation between the control signal at the gate terminal and the aging paths. Therefore, the aging dependence among different devices is necessary for SP analysis.

### 2.3. Impact of Aging Dependence

**Figure 4:**
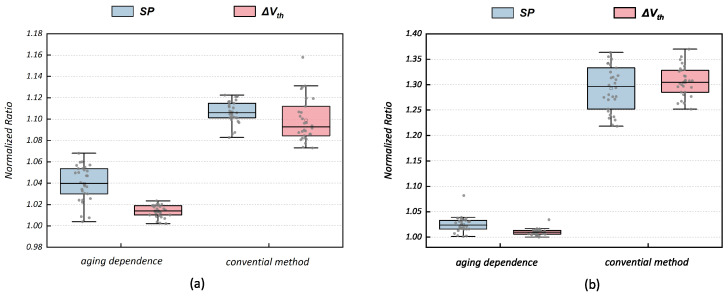
NBTI normalized *SP* and Δ *V*_th_ degradation of *MP*5 (**a**) and *MP*10 (**b**) in full adder based on the conventional algorithm and the method considering aging dependence.

To further quantify the impact of aging dependence, the transistors MP5 and MP10 discussed above are chosen as examples. Several testing workloads (different sets of primary input signal waveform) are adopted at transistor-level simulations based on the commercial SPICE simulation tool [22]. Through transient reliability simulations, the aging result obtained is set to be golden. Referring to the duty cycle of input signals and utilizing Equations (3)–(7), we compute analytical results for both the conventional method and the approach that considers dependencies individually. All SP outcomes will be converted to normalized results based on the simulation results. As illustrated in Figure 4, the outcomes yielded by the conventional method appear consistently over-pessimistic across varying workloads. This phenomenon is particularly pronounced in the case of *MP*10, which needs to be discussed under more complex logical conditions.

After compiling statistics for all testing results, it is observed that the average SP errors for the conventional algorithm are +10.6% on *MP*5 and +29.3% on *MP*10. In contrast, the analytical model, which considers the aging dependence, exhibits lower errors at +4.0% on *MP*5 and +2.5% on *MP*10. Then, Δ *V*_th_ are calculated based on the SP results above, where the used aging model will be given in Section 4.1. As shown in Figure 4, the average Δ *V*_th_ errors of the conventional algorithm are +9.9% on *MP*5 and +30.6% on *MP*10. The values for the analytical model considering the aging dependence are +1.4% on *MP*5 and +1.0% on *MP*10. Additionally, although we only discussed *MP*5 and *MP*10 for NBTI, most other PMOS and NMOS transistors for BTI aging are in similar situations. In other words, this issue is prevalent with the degradation evaluation of all transistors in the circuit.

The above comparison shows that the conventional algorithm will induce overestimated results at the cell level. Furthermore, the overestimation will induce the tight constraints of the aging guardband at the circuit level, and reliability-aware circuit design will sacrifice more performance (relevant experimental support will be given in Section 4.4). The analytical model considering the aging dependence gives more accurate outcomes and significantly reduces overestimation.

## 3. Proposed Method

As mentioned above, exploring a new analysis model to catch the aging dependence is crucial, especially in the cells like the full adder and other more complex block cells. This section will introduce our method, which is based on logical resolving.

First of all, the problem will be converted into the equivalent description: under the assumption that all the inputs of gate cells are independent, and all logical combinations which will induce BTI stress of each transistor need to be figured out. And then, the final SP of BTI effect is derived by all combinations (note that, when these logical events are independent, the results equal to the accumulating probabilities of all logical events).

Equivalently, the problem can be treated as a typical Boolean satisfiability problem (SAT) [23], with the Boolean formulas representing the BTI stress conditions of transistors. To enumerate all possible solutions to SAT problems, the concept of all solutions SAT [24] is adapted and can be addressed by traversing the binary decision diagram (BDD) structure.

Again, take NBTI as an example. As displayed in Algorithm 1, the proposed method mainly consists of four stages. The required inputs contain the transistor netlists of cells and the duty cycle of cell inputs. First, the proposed method will parse the SPICE netlist of gate cells and convert the transistors network to an undirected graph (Algorithm 1 lines 1–7). All intermediate signals and supply voltage “VDD” vertex are presented as vertexes of the graph. The transistors between the two neighboring vertexes will be converted to the nets without direction. Second, we will obtain all paths between the “VDD” vertex and other vertexes through breadth-first search (BFS) traversal. The relating signal can be charged to logic “1” when any path is conducted. In this way, the corresponding Boolean function can be initiated by the transistor paths (Algorithm 2 lines 1–11). Third, the proposed algorithm will obtain the stress function of each PMOS transistor and convert it into a BDD structure (according to the relationship in Equation (1)), which is based on the primary input variables (Algorithm 3 lines 1–7). At last, solving the SAT of the stress function would be equivalent to finding all the paths that end with logical “1” in the BDD structure.
**Algorithm 1** SP calculation-based logical topology**Input:** cell subcircuit, 
PL/PH
 of input signals
**Output:**

fetSP
: SP of every device
  1: **for** each signal *s* except inputs **do**  2:    convert *s* to be vertex *v* of *g*  3:    **for** each transistor *t* which links to *s* **do**  4:        convert *t* to be graph net *n* of *g*  5:        connect *s* and *t*  6:    **end for**  7: **end for**  8: get all signal Boolean function using Algorithm 2  9: build all BDD structure for each device using Algorithm 310:**for** BDD node citcuitBDD of each device **do**11:     find all true logic path btiPathSet
12:     by BFS on circuitBDD
13:     **for** each path *p* in btiPathSet **do**14:      get logic probability pathSP of *p*15:      
SP = SP

                     +           
pathSP


16:   **end for**17:** end for**


**Algorithm 2** Obtain all signal Boolean function**Input:** graph *g*
**Output:** Boolean function of each signal
  1:**for** each vertex *v* of pull-up network **do**  2:    symbol Boolean function of *v* as BF = 0  3:    find all path pathSet between VDD and *v* by BFS  4:    **for** each path *p* of pathSet **do**  5:        set path function to be pf = 1  6:        **for** each mosfet *m* in path *p* **do**  7:            
pf = pf and not(gate signal of *m*)  8:        **end for**  9:        
BF = BF or pf
10:   **end for**11:**end for**12:**for** each vertex *v* of pull-down network **do**13:    symbol Boolean function of *v* as BF = 114:    find all path pathSet between VSS and *v* by BFS15:    **for** each path *p* of pathSet **do**16:        set path function to be pf = 017:        **for** each mosfet *m* in path *p* **do**18:            
pf = not ( pf or (gate signal of *m*))19:        **end for**20:        
BF = BF and pf
21:    **end for**22:**end for**


**Algorithm 3** Build BDD structure**Input:** graph *g*, cell subcircuit, signal Boolean function
**Output:** BDD node for stress condition of each device
  1:**for** each PMOS pm in cell subcircuit **do**  2:   symbol NBTI stress function of pm as SF
  3:   get Boolean function gateBF of pm ’s gate vertex  4:   get Boolean function sourceBF of pm ’s source vertex  5:   get Boolean function drainBF of pm ’s drain vertex  6:   
SF = not ( gateBF ) and ( sourceBF or drainBF )  7:   convert SF to BDD node of circuitBDD
  8:**end for**  9:**for** each NMOS nm in cell subcircuit **do**10:   symbol PBTI stress function of nm as SF
11:   get Boolean function gateBF of nm ’s gate vertex12:   get Boolean function sourceBF of nm ’s source vertex13:   get Boolean function drainBF of nm ’s drain vertex14:   
SF = gateBF and ( not ( sourceBF ) or not ( drainBF ) )15:   convert SF to BDD node of circuitBDD
16:**end for**


It is noteworthy that BDD is constructed by recursively partitioning the decision tree into paths representing logical false and logical true. At each node, a variable is chosen, and the graph is divided into two branches: one representing the variable as false and the other as true. This process continues recursively until reaching terminal nodes. Therefore, the structure of the BDD inherently ensures that all resolved logical combinations are mutually exclusive.

As a result, our method will obtain the final SP value by directly multiplying the signal probability in each logical path and accumulating all path conducting probabilities (Algorithm 1 lines 10–17).

**Figure 5:**
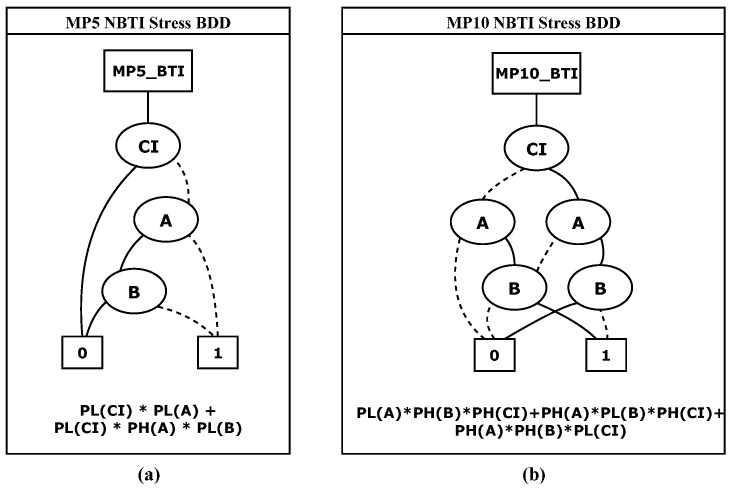
BDD structure of BTI stress function and the final result for (**a**) *MP*5 and (**b**) *MP*10, where the endpoint “0” presents that the logic combinations will not cause degradation, and endpoint “1” is the opposite.

In summary, the SP evaluation for std-cell is realized by abstracting a logical relationship from the SPICE netlist and solving it as a typical SAT problem. The error induced by aging dependence will be avoided. Corresponding to the previous conclusions in Section 2, Figure 5 illustrates the stress BDD structures and the result of *MP*5 and *MP*10. The solid net presenting that signal takes a value of logic “1” and logic “0” for the dotted net. All paths listed end with logic “1” will be obtained by traversing these two logic branch diagrams. By summarizing all paths corresponding probability, the final SP equations are consistent with aforementioned results in Equations (4) and (7). Note that the proposed method treats pull-up and pull-down networks separately for NBTI and PBTI. So, the proposed method can also handle non-mirror structural cells other than full adder. 

## 4. Results and Discussion

To employ SPICE aging simulation and ensure further verification, it is necessary to determine and calibrate the aging model. This section will first calibrate the aging model used. Considering that the SP evaluation forms the basis for both aging critical path analysis and aging-aware libraries characterization, as illustrated in Figure 2; we will implement these two processes separately to verify that our method can enhance the accuracy for path delay analysis and aging-aware libraries generation. The conventional analytical methods [9,16] are chosen as the comparisons. Moreover, the circuit performance improvement for the aging-aware synthesis flow will be demonstrated. In our experiment, all std-cells are from the Nangate 45 nm Open Cell Library [25] and the high-k 45 nm Predictive Technology Model (PTM) [7] are utilized (which correspond to the process node of selected cell library). The commercial SPICE simulation tool [22] and aging model interface [26] are employed to obtain aging simulation results. The CUDD package [27] is used to build the BDD manager and all algorithms are implemented in C++ language.

### 4.1. Aging Model Calibration

For the model calibration, we refer to the aging model from the commercial aging simulation tool [11] after calibration with experimental data from the work [28]. The proposed method is independent of the aging model. Because SP is a factor related to the physical mechanism of BTI aging, the proposed method can apply to other more advanced technology and more complex aging models.

**Figure 6:**
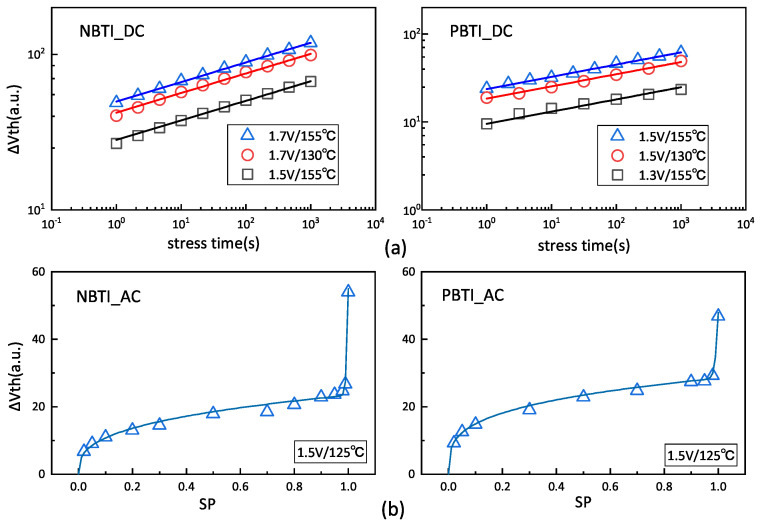
Calibrations of the experimental data for aging model, all the experiment data come from [28]: (**a**) the data for NBTI and PBTI aging under DC stress and model fitting; and (**b**) the data for NBTI and PBTI aging under AC stress and model fitting, where the AC stress time equals 1000 s.

As observed in Figure 6a,b, the fitted model matches the experimental data on both DC (i.e., the stress voltage will remain constant and there is no recovery stage) and AC (i.e., the stress voltage will switch and contain both stress and recover stages) stress conditions. Here, the form of the aging model is given:

(8) $$\begin{array}{c} \left\{\begin{array}{c} \begin{array}{c} \Delta V_{\mathrm{th}}\left(BTI\text{\_}DC\right)=R_{a}\times e^{G\times \left|V_{gs}\right|}\times e^{-\frac{Ea}{KT}}\times t_{stress}^{n}\\ \Delta V_{\mathrm{th}}\left(BTI\text{\_}AC\right)=\Delta V_{\mathrm{th}}\left(BTI\text{\_}DC\right)\times \left(A\times SP^{\alpha }+B\times \log_{10}\left(1+SP^{\beta }\right)\right) \end{array} \end{array}\right. \end{array}$$

where $R_{a}$ is a pre-factor; *G* is a voltage dependency; Ea is a temperature dependency; *K* is the Boltzmann’s constant (8.617 × $10^{-5}$ eV/K); *T* is the channel temperature in Kelvin; and α and β are fitting coefficients. It should be noted that, while the aging model does not account for the different sources of fluctuations, it can be easily extended. This extension involves considering the fluctuations within the aging model or incorporating their effects at the circuit level [29]. As these fluctuations have a minimal impact on the aging time during device operation, our proposed stress probability extraction method remains effective.

### 4.2. Path-Level Analysis

**Figure 7:**
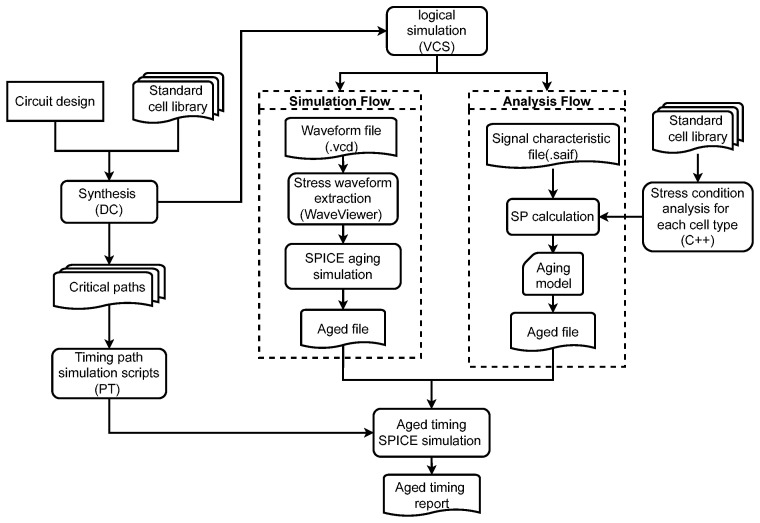
Evaluation flow of aging-aware STA for the critical paths.

To verify the accuracy of the proposed method, the BTI aging timing analysis framework for circuit paths will be introduced in this subsection. The chosen designs are from the ISCAS’85 benchmark [30]. As depicted in Figure 7, the circuit design is synthesized using design compiler (DC). Subsequently, sets of random testbenches are established, and the Verilog compiled simulator (VCS) tool is employed for logical simulation (as the ISCAS’85 benchmark circuits [30] are some general computing modules, it is reasonable to randomly generate arbitrary input signals). Following this, the switching activity interchange format (SAIF) document and the waveform file in the value change dump (VCD) standard format are obtained.

The different processing methods can be divided into two flows. The SP and Δ *V*_th_ can be obtained by reliability simulation and the simulation result will be treated as the baseline of comparison. For the analysis flow, the inputs are the cell library, SAIF file. and path sets. First, all the combinational cells in the specified std-cell library will be analyzed, and all cell degradation relations will be stored in memory. It should be emphasized that the process only needs to be executed once for one library. Then, the PL and PH of each signal are determined by parsing the SAIF file, and the analysis result can be obtained immediately. After the two aforementioned steps, the threshold voltage shifting is calculated based on the aging model. Finally, we apply primer time (PT) to generate the SPICE decks of critical paths, and all aged path delays can be simulated by considering the aging result records. The entire framework is automatically accomplished by python and tool control language (TCL) scripts.

All Nangate 45 open-cell library cells are included for the synthesis of all circuit designs. Experiments are tested with an AMD Ryzen 9 5900X machine (12 cores/24 threads @3.8 GHz) and a SPICE simulation tool [22] is set to run on 16 threads. The top 50 critical paths of each design are chosen for analysis, and selection criteria is based on the fresh timing report. Although there is a situation where the post-degradation critical path and the fresh critical path switch [31], our experiments are generic for all paths and this paper mainly focus on the aged timing accuracy in this paper rather than the correctness of path selection. Therefore, we simplify it here and only discuss the accuracy of path degradation analysis.

All the results are listed in Table 1, where the MAX, MIN, and AVG represent the maximum, minimum, and average delay/errors among all selected paths in every design. As stated in the “aged delay” columns, the results of the conventional analytical methods are approximately +47 ps. And, stability is not guaranteed in different design examples, like the lower bound of design C880 exceeding 133 ps and the upper bound of design C6288 exceeding 126 ps. For our approach, all examples have errors ranging from −3.1 ps to +44.3 ps. And, most of our errors are within 10 ps and the average error is reduced to +12.9 ps. To compare the gap with the simulation results, the relative error is evaluated to measure the quality of the results. The “relative error” columns of Table 1 show the relative error value of all 50 paths in every design. This indicates that all relative error results of the conventional analytical methods [9,16] will be greater, and the total relative error will exceed 2.3%. In contrast, most of our results are within the ±1% relative error range, and the total average relative error is only +0.53%. Although our result may lead to some lesser degree of optimistic estimation, these all happen in the lower bound. The impact will increase the amount of degraded critical paths being considered by a few degrees, and the error is within 0.5%. To avoid such underestimation, a pessimistic assumption can be made by considering the aging conditions at the highest voltage (FastFast corner) and the highest temperature (SlowSlow corner) among all corners (for the Nangate 45 nm open-cell library [25], the SlowSlow process corner conditions are 0.95 v and 125 °C, while the FastFast process corner conditions are 1.25 v and 0 °C). This ensures that the transistor aging results are pessimistic relative to the actual operating environment of the circuit.

For the running time, as shown in the “Run time” columns of Table 1, the value only considers the time consumption caused by calculating the degradation result of all 50 paths. From the overall results, the runtime of our method is nearly 378 times faster than the simulation flow.

### 4.3. Aging-Aware Std-Cell Library Characterization

Apart from the above-discussed accurate aging path delay analysis with the proposed method, the improved aging-aware library characterization will be discussed in this subsection. Due to the reason that degeneration would vary with workload changes [9,16], the instances with the same type in the circuit netlist will be aged differently. For the aging timing analysis of the entire circuit, the efficient method is to generate aging-aware std-cell libraries under different aging stress scenarios (i.e., various combinations of voltage, temperature, and the duty cycle of inputs). We will give the cell-level comparison results to demonstrate that the aging-aware characterization flow with the proposed method can create more accurate timing results for the std-cell library.

**Figure 8:**
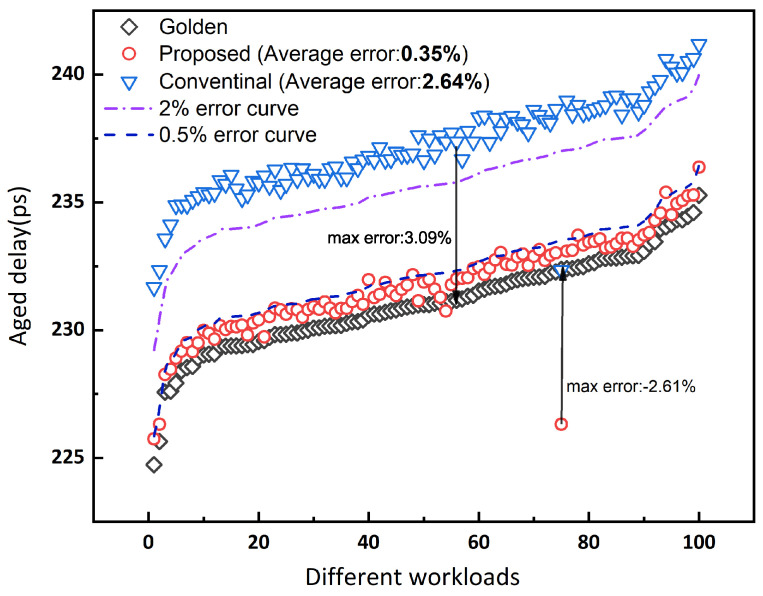
The aged delay of FA_X1 under different workloads, where the random waveform for workloads is generated by applying the perjitter command [32] in commercial SPICE simulation tool [22].

Regarding the accuracy of aging timing results in the characterized libraries, the FA_X1 cell (which is the most complex combinational logic std-cell in Nangate 45 nm open cell library) is selected for the experiment under different aging scenarios. To validate the fact that the proposed method can effectively capture the relationship between aging delay and workload, 100 sets of aging workload are randomly generated and applied to the FA_X1 cell (the stress temperature was set to 125 °C, the stress voltage was set to 0.95 V, and the aging time was set to 10 years). This result is depicted in Figure 8. The upper blue data points show that the results of the conventional analytical method are almost over-pessimistic; the lower red data points show that our method captures the workload dependence with a minor error. On average, the delay error of the conventional analytical method is 2.64%, while the error is only 0.35% for the proposed method.

Although the worst case has a max error of −2.61%, this is mainly caused by the strong correlation between input signals.

The vast majority of our results have a relative difference of less than 0.5%. In contrast, almost all conventional analytical results have an error of more than 2%.

Our method with more accurate results enables the mitigation of the pessimistic prediction at the cell level compared with the conventional analytical approach. Moreover, the aging library characterization process combined with the proposed method will lighten the over-pessimism evaluation of timing degradation. It is noteworthy that the circuit is composed of the bounds of cells. The final error at the circuit level would be greater than the effect of a single cell.

### 4.4. Aging-Aware Synthesis

**Figure 9:**
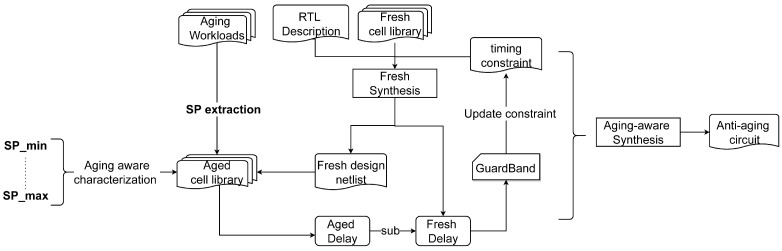
The analysis flow of the timing guardband and aging-aware synthesis.

In addition to accurate predictions for path analysis and aging-aware characterization, the circuit-level reliability design flow incorporating our approach will be described in this subsection. We adopt the concept of the timing guardband [18], which is illustrated by Figure 9.

As illustrated on the left side of Figure 9, the range of $SP_{min}$ and $SP_{max}$ is determined for aging-aware library characterization. Subsequently, all instances in the fresh netlist are analyzed to obtain stress probabilities. Following that, the aged netlists are inferred by changing the cell type of instances to aged ones, and the aged timing delay of aged netlists is analyzed based on aging-aware libraries. To allocate the design margin in advance, the timing guardband is calculated as the difference between the aged delay and fresh delay. After a new round of the circuit synthesis process by tightening the timing constraints, the anti-aging netlist can be obtained.

SP evaluation for the workloads and characterization is the basis of the reliability-aware synthesis flow. Pessimistic predictions brought by conventional analytical methods will lead to extra design costs for the circuit and increase the convergence difficulty. To verify this trend, we employ the flow of aging-aware library characterization in the work [18], and the SP values of pull-up and pull-down networks are discretely sampled in the interval [0.1, 1.0] with a step size of 0.1. In other words, 100 sets of aging libraries will be characterized. And then, the SPICE netlists of corresponding aged cells will be updated based on the combination of SP, aging model and fresh BSIM model. For example, the aged cell XOR_X1_n0.1_p0.2 represents that all NMOS in XOR_X1 cell is under stress with 10% SP, and all PMOS are under stress with 20% SP. The SP value between sample points will be rounded up to the upper value. All of the cells in the Nangate 45 nm library are included, and the various sets of aging libraries are characterized first. Then, the SP obtained by the proposed and conventional analytical method will serve as updated guidance for the aged netlist. The AES_CORE from IWLS’05 benchmark [33] is chosen as the test circuit. After synthesis, the corresponding netlist contains 23 thousand std-cells. The aging workloads come from the Yosys testing suit [34], and the SAIF result after 280 encryption operations is used as the input of the SP evaluation algorithm.

**Figure 10:**
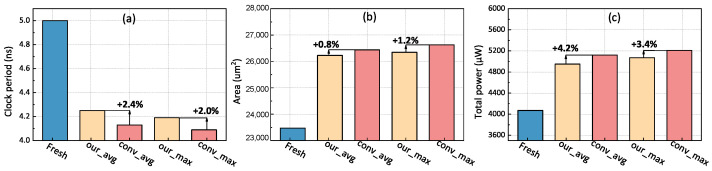
Performance, power, and area of aging-aware synthesis result comparison between the proposed and conventional analytical [16] method: (**a**) clock period; (**b**) synthesis area; and (**c**) total power consumption.

We experiment with the average and maximum SP values, as indicated in Figure 10. In other words, the average and maximum values of the SP of all devices in the pull-up and pull-down network are taken as the SP value, respectively. Our method will only take 8 s to analyze the input, calculate the degradation results of all internal devices, and finally output the degraded netlist information.

To fairly quantify the design cost, all parameters except the clock period remain the same. In addition, the worst negative slack is guaranteed to be 0. The results shown in Figure 10a explain that the conventional analytical method will require a lower clock period, which means the timing constraint is stricter, tightening up more 2.4% and 2.0% timing requirements under average and maximum conditions. Furthermore, the tighter design constraints will induce a greater area and power consumption, as illustrated in Figure 10b,c. This explains that a higher aging evaluation will tighten up the timing constraint of the initial design to reserve a greater design margin. As a result, this will sacrifice at least 0.8% and 3.4% in terms of area and power, respectively, to compensate for the effects of these errors.

In summary, except for the path-level reliability analysis, the proposed method can achieve greater accuracy for the reliability-aware library characterization. Furthermore, combining reliability-aware design flow at the circuit level will avoid design costs and complex design convergence. Note that the proposed method can handle more complex circuits like macroblocks and smaller-scale unit circuits, in which the aging dependence is evident.

## 5. Conclusions

This paper proposes an accurate and efficient aging-aware stress analysis flow. A logical-resolving method of SP evaluation for BTI aging is proposed, and the aging dependence among aging devices is carefully considered for the first time. The accuracy is evaluated at the path level, which will guide the proper degradation path analysis and selection. Moreover, the proposed method is embedded with commercial EDA tools to establish the aging-aware library characterization flow. The degree of over-pessimism will be reduced for reliable library generation under the same aging conditions. Furthermore, the proposed method is beneficial in lightening the excessive pessimism of design margins for the aging-aware synthesis flow. This work shows that aging dependence is essential for accurate BTI aging analysis in digital circuit, and the proposed method is effective in solving it. Experimental results show that the proposed method can maintain an average error of aging delay at about 0.5% and achieve this hundreds of times faster than the SPICE aging simulation. In conclusion, the accurate results from the proposed approach can benefit the aging-aware STA flow, hence giving constructive guidance in the early stage of circuit design for reliability.

## Figures and Tables

**Table 1:** Aged path delay of benchmark circuits.

Design		Fresh Delay	Aged Delay (ps)				Relative Error			Run Time (s)			
			Simulation	Conventional Analysis [16]	Floating Model [9]	Proposed	Conventional Analysis [16]	Floating Model [9]	Proposed	Simulation	Conventional Analysis [16]	Floating Model [9]	Proposed
C432	MAX MIN AVG	1376.1 1268 1338.2	1564.1 1344.6 1449.3	1600.1 (+36) 1349.5 (+4.9) 1472.1 (+22.8)	1600.3 (+36.2) 1349.5 (+4.9) 1472.2 (+22.9)	1570.3 (+6.2) 1345.7 (+1.1) 1453.5 (+4.2)	+2.7% +0.40% +1.55%	+2.7% +0.40% +1.55%	+0.80% +0.10% +0.29%	557.89	13.91	26.23	16.24
C499	MAX MIN AVG	1200.2 1078.2 1118.5	1299.6 1202.1 1238	1381.9 (+82.3) 1223.9 (+21.8) 1271.3 (+33.3)	1380.1 (+80.5) 1223.9 (+21.8) 1270.5 (+32.5)	1343.9 (+44.3) 1199.7 (−2.4) 1242.9 (+4.9)	+6.5% +1.5% +2.65%	+6.3% +1.5% +2.60%	+3.5% −0.4% +0.37%	1058.47	4.94	11.01	7.41
C880	MAX MIN AVG	1286.5 707.9 1074.3	1564.8 727.2 1246.9	1601.4 (+36.6) 860.76 (+133.6) 1291.11 (+44.2)	1601.4 (+36.6) 858.54 (+131.3) 1290.87 (+43.9)	1584.8 (+20) 727.17 (−0.03) 1246.9 (+0)	+18.4% +1.8% +4.2%	+18.1% +1.8% +4.1%	+14.1% +0% +2.3%	949	6.20	16.58	7.43
C1355	MAX MIN AVG	1268.8 1142.6 1223.3	1432 1169.3 1375.9	1473.2 (+41.2) 1177 (+7.7) 1413.1 (+37.2)	1472.2 (+40.2) 1177 (+7.7) 1412.452 (+36.6)	1434.8 (+2.8) 1172.1 (+2.8) 1381.4 (+5.5)	+6.5% +1.5% +2.6%	+4.2% +0.7% +2.6%	+2.5% +0% +0.4%	1138.95	6.54	10.74	8.18
C1908	MAX MIN AVG	1203.4 1124.1 1165.2	1366.1 1235.7 1235.1	1428.5 (+62.4) 1257.7 (+22.1) 1342.1 (+107.3)	1428.5 (+62.4) 1257.8 (+22) 1342.4 (+107)	1402.2 (+36.1) 1239.4 (+3.7) 1316.1 (+81)	+4.9% +1.6% +2.4%	+4.9% +1.7% +2.5%	+2.9% −0.4% +0.45	894.69	3.95	7.27	4.94
C2670	MAX MIN AVG	903.9 762.4 835.2	1043.5 800.7 922.2	1064.7 (+21.2) 805.7 (+5) 938.6 (+16.4)	1064.7 (+21.2) 805.7 (+5) 938.3 (+16.1)	1052.7 (+9.2) 801.6 (+0.9) 926.4 (+4.2)	+3% +0.4% +1.7%	+3% +0.03% +1.7%	+0.9% −0.1% +0.4%	3525.3	11.54	16.15	12.93
C3540	MAX MIN AVG	1762.3 1281.9 1541.9	2055.9 1374.6 1755.2	2100.1 (+44.2) 1380.4 (+5.8) 1794.7 (+39.5)	2097.1 (+41.2) 1380.4 (+5.8) 1793.97 (+38.8)	2052.8 (−3.1) 1376.6 (+2) 1760.6 (+5.4)	+2.7% +0.4% +2.2%	+2.7% +0.4% +2.2%	+0.8% −0.2% +0.3%	12,656.94	7.72	18.53	11.58
C5315	MAX MIN AVG	1371.5 1220.9 1298.2	1582.1 1439.4 1495.2	1616.8 (+34.7) 1469.4 (+30.1) 1533.8 (+38.7)	1616.8 (+34.7) 1469.5 (+30.1) 1533.9 (+38.7)	1582.7 (+0.6) 1442.1 (+2.7) 1497.2 (+2)	+3.2% +1% +2.6%	+3.3% +1% +2.6%	+0.8% −0.1% +0.1%	6025.00	11.99	18.16	17.99
C6288	MAX MIN AVG	3853.1 3611.4 3729.0	4381.7 3949.0 4205.5	4513.8 (+132.1) 4033.1 (+84.1) 4308.8 (+103.3)	4508.5 (+126.8) 4034.0 (+85) 4310.1 (+104.6)	4386.4 (+4.7) 3956.3 (+7.3) 4222.5 (+17)	+3.1% +1.9% +2.4%	+3.1% +1.8% +2.2%	+0.8% +0.1% +0.4%	8248.9	22.25	47.46	33.36
C7552	MAX MIN AVG	1459 1307.4 1380.0	1668.2 1359.3 1538.5	1709.7 (+41.5) 1361.4 (+2.1) 1569.3 (+30.8)	1709.5 (+41.3) 1361.4 (+2.1) 1569.2 (+30.7)	1676.6 (+8.4) 1360 (+0.7) 1543.5 (+5)	+2.8% +0% +1.9%	+3.0% +0.4% +1.7%	+1.1% −0.5% +0.3%	14,160.03	11.604	19.53	14.505
Total average		-	-	+47.4	+47.2	**+12.9**	+2.42%	+2.37%	**+0.53%**	4921.52	**10.06**	19.17	13.46

## Data Availability

Data are contained within the article.
